# Supplementary material for: HOTAIR Participation in Glycolysis and Glutaminolysis Through Lactate and Glutamate Production in Colorectal Cancer
Source: Cells. 2025 Mar 6;14(5):388. doi: 10.3390/cells14050388 (PMC11898799; doi:10.3390/cells14050388)
Supplement: Supplementary file 1 [file cells-14-00388-s001.zip › cells-3477114-supplementary/SUPPLEMENTARY FIGURES.pdf]

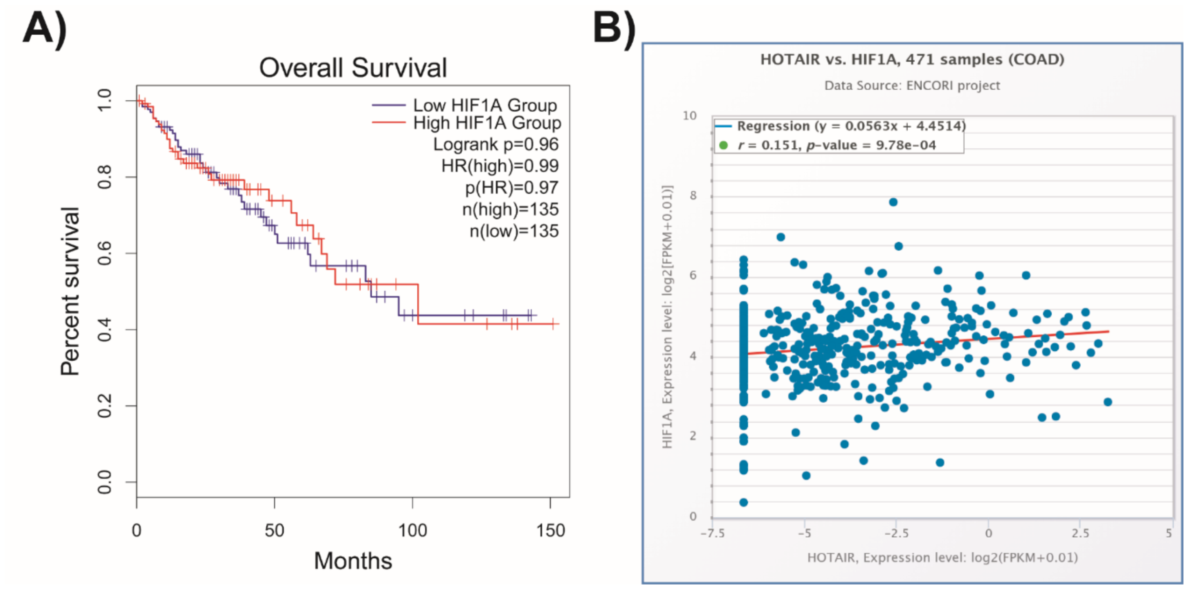

SUPPLEMENTARY FIGURE S1. HIF-1α expression in tumor tissue. A) Kaplan-Meier survival curve of 132 colorectal cancer patients overexpressing HOTAIR versus 130 patients with low HOTAIR expression (GEPIA 2). **B)** Correlation of HOTAIR/HIF-1α expression in colorectal cancer patient samples.

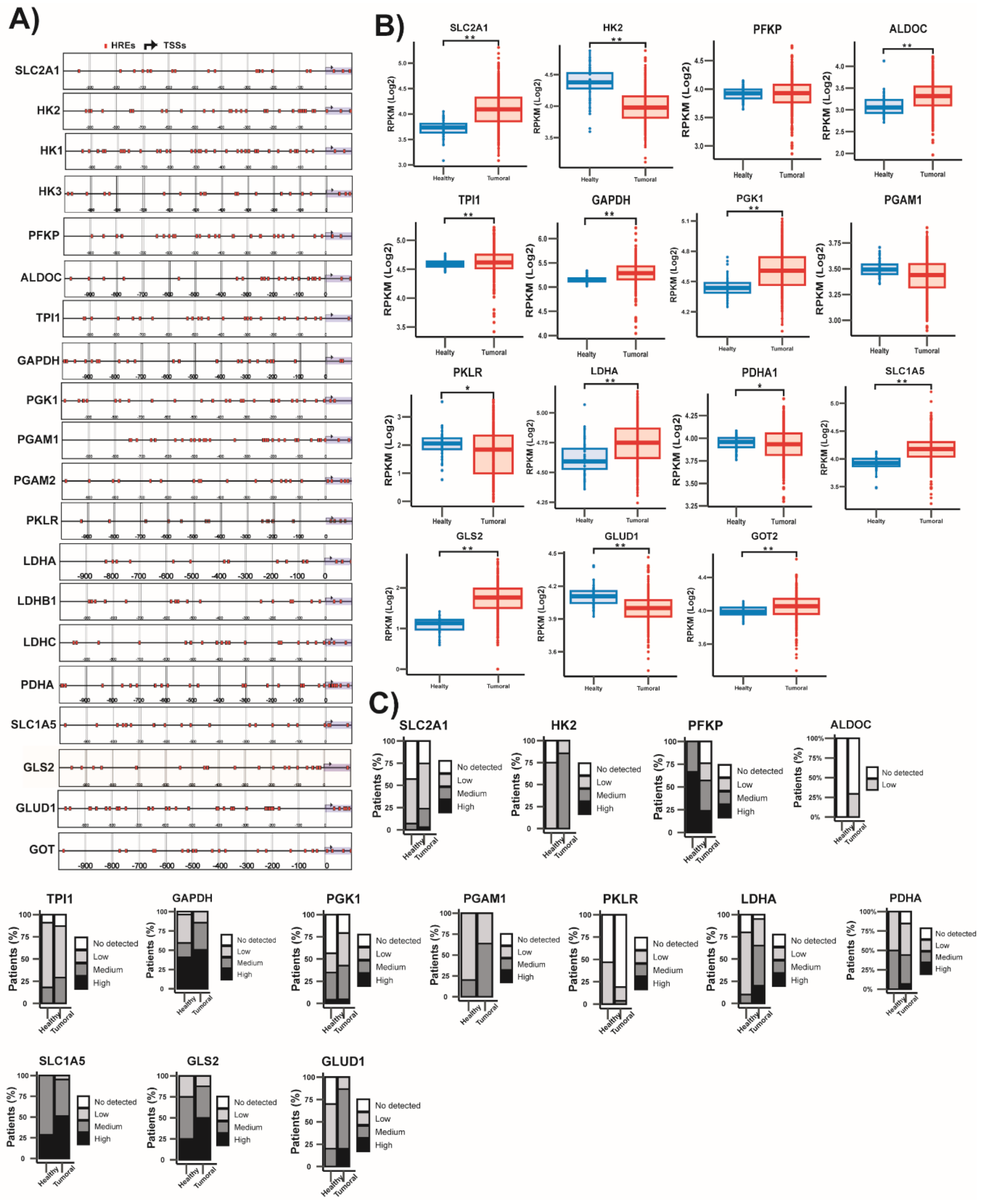

SUPPLEMENTARY FIGURE S2. Bioinformatic analysis of the regulatory effect of HIF-1α on metabolic enzyme expression. A) JASPAR prediction of hypoxia response elements (HREs) present in metabolic enzyme promoters (p-value 0.01). **B)** Metabolic enzyme expression levels in 471 tumor tissue samples versus 41 non-tumor tissue samples (TCGA). **C)** Immunohistochemistry of metabolic enzymes in non-tumor vs. tumor tissue.

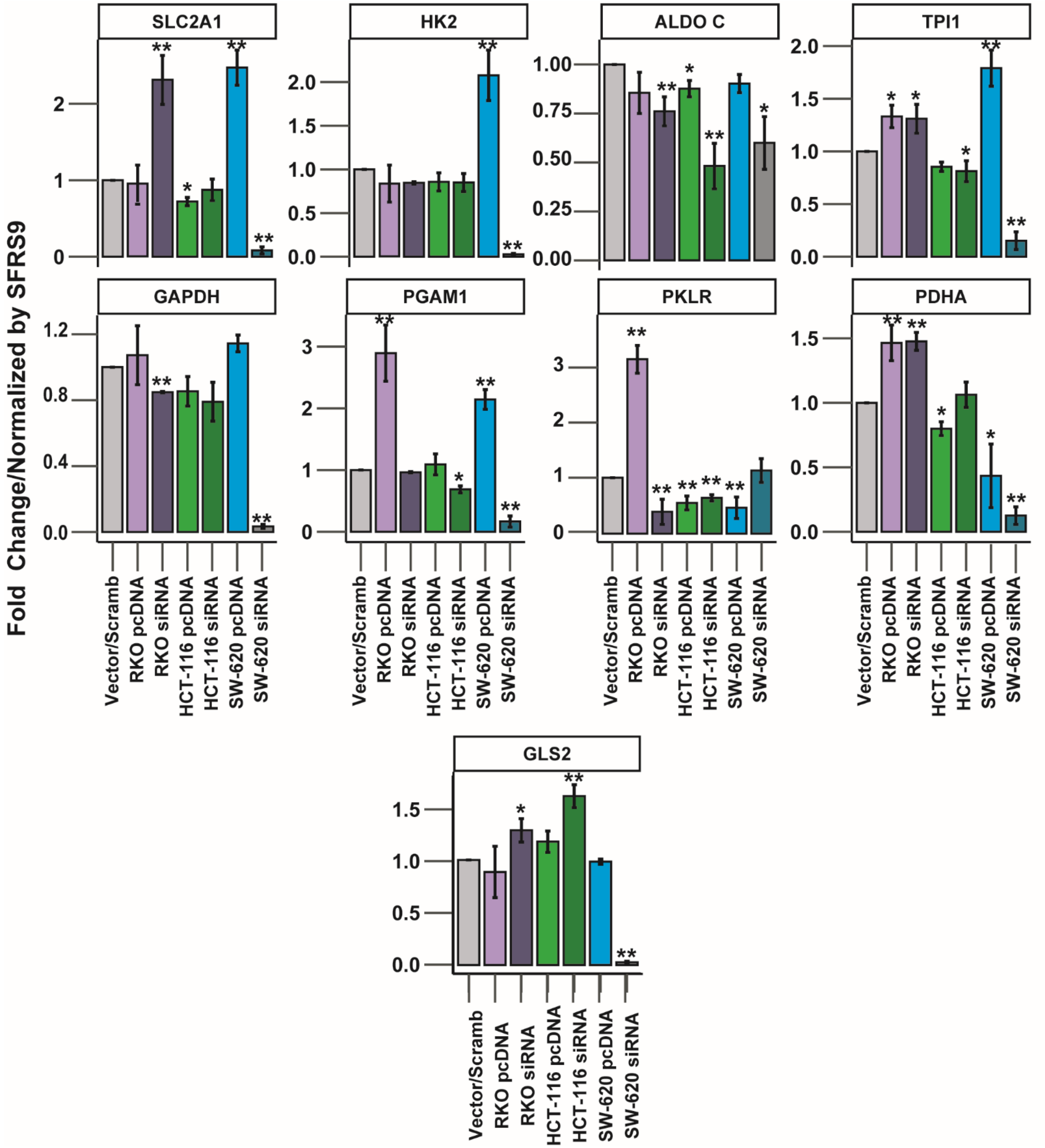

SUPPLEMENTARY FIGURE S3. Expression levels of glycolytic and glutaminolytic enzymes regulated by HOTAIR in the differential expression model. Data are presented as mean ± SD. *p < 0.05, **p < 0.005.

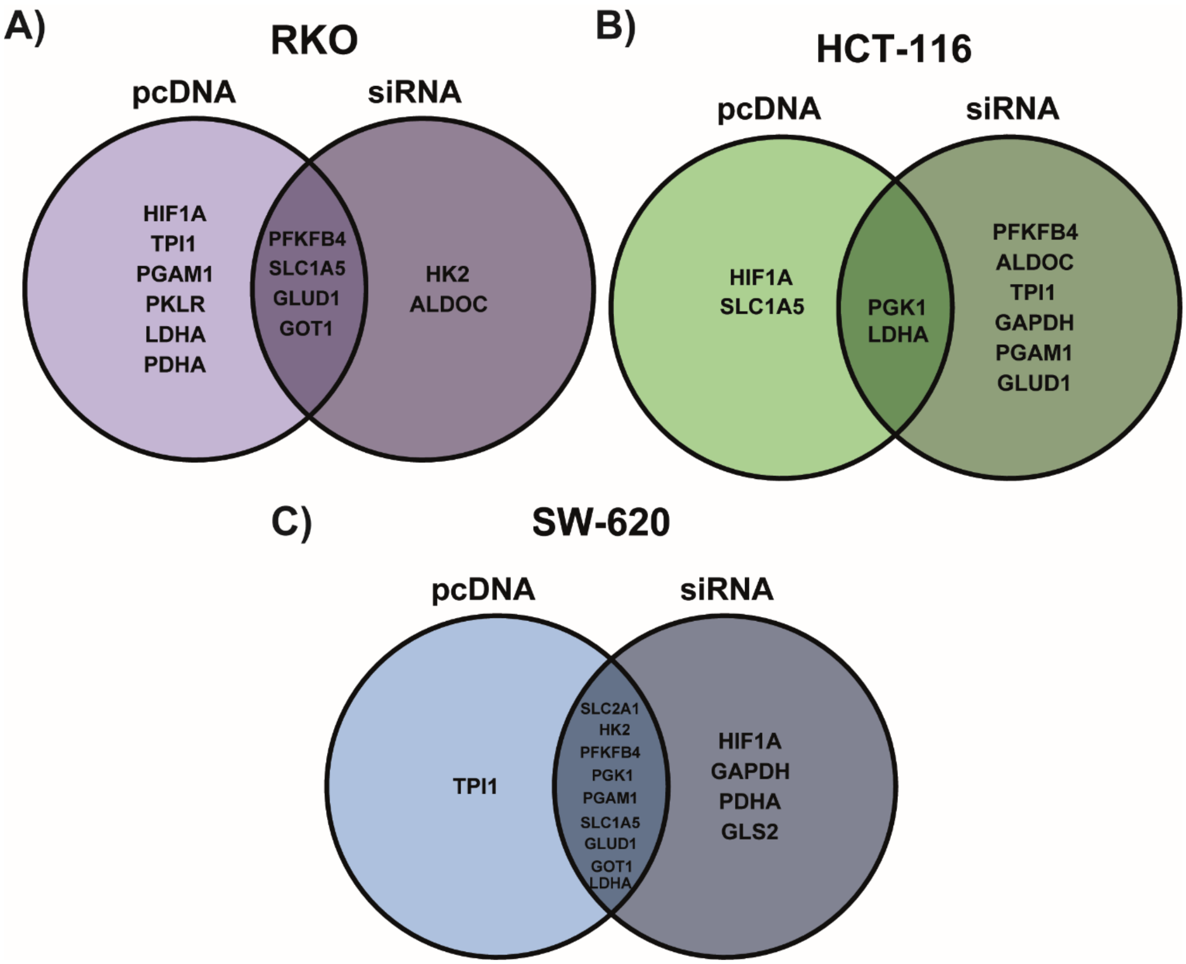

SUPPLEMENTARY FIGURE S4. HOTAIR-regulated metabolic enzymes in three colorectal cancer models. A) Venn diagram of differentially expressed enzymes following modification of HOTAIR expression in RKO cells. **B)** Venn diagram of differentially expressed enzymes following modification of HOTAIR expression in HCT-116 cells. **C)** Venn diagram of differentially expressed enzymes following modification of HOTAIR expression in SW-620 cells.

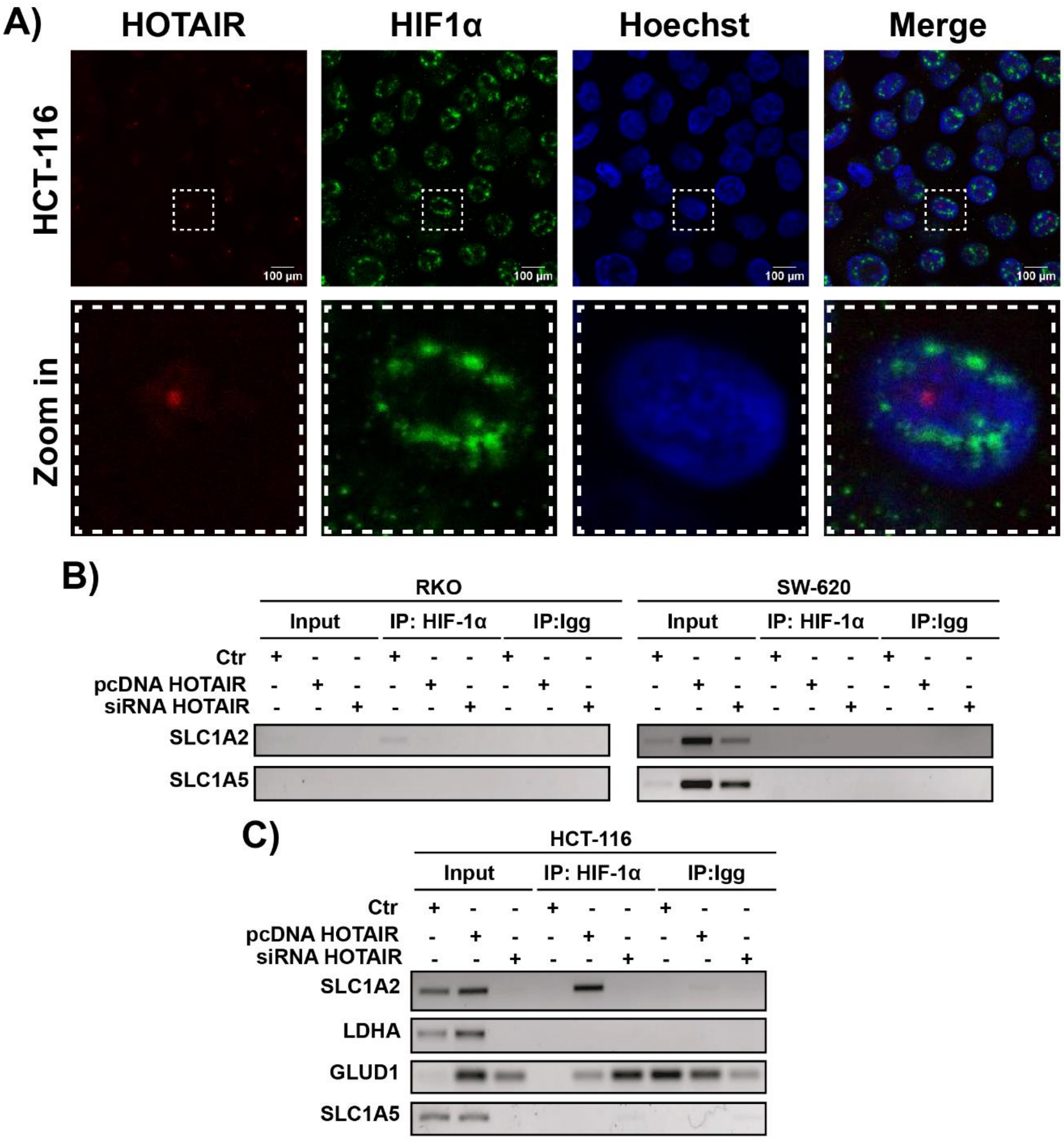

SUPPLEMENTARY FIGURE S5. Cellular distribution of HOTAIR/HIF-1α. A) Colocalization of HOTAIR and HIF-1α in HCT-116 cells. **B)** Positioning of HIF-1α at the SLC1A2 and SLC1A5 promoters by Chi-P assay in RKO and SW-620 cells. **C)** Positioning of HIF-1α at the promoters of metabolic enzymes by ChiP assay in HCT-116 cells.

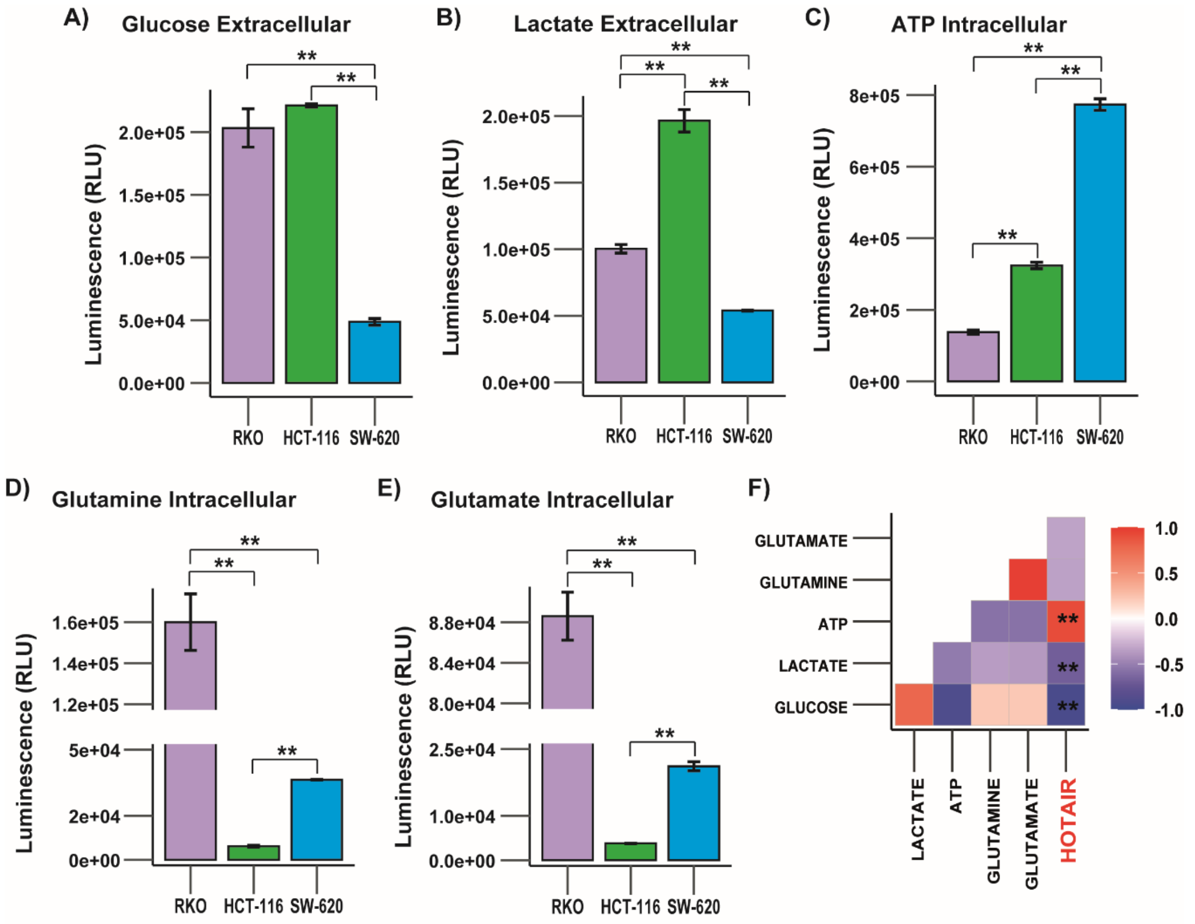

SUPPLEMENTARY FIGURE S6. Basal metabolism of colorectal cancer cells. A) Glucose uptake in colorectal cancer cells. **B)** Lactate production in colorectal cancer cells. **C)** ATP production in colorectal cancer cells. **D)** Glutamine uptake in colorectal cancer cells. **E)** Glutamate production in colorectal cancer cells. **F)** Correlation Matrix of HOTAIR and glycolysis/glutaminolysis metabolites in colorectal cancer cell lines. Data are presented as mean ± SD. $^*p < 0.05$, $^{**}p < 0.005$.
