# Supplementary figures and images for: HOTAIR Participation in Glycolysis and Glutaminolysis Through Lactate and Glutamate Production in Colorectal Cancer
Source: Cells. 2025 Mar 6;14(5):388. doi: 10.3390/cells14050388 (PMC11898799; doi:10.3390/cells14050388)

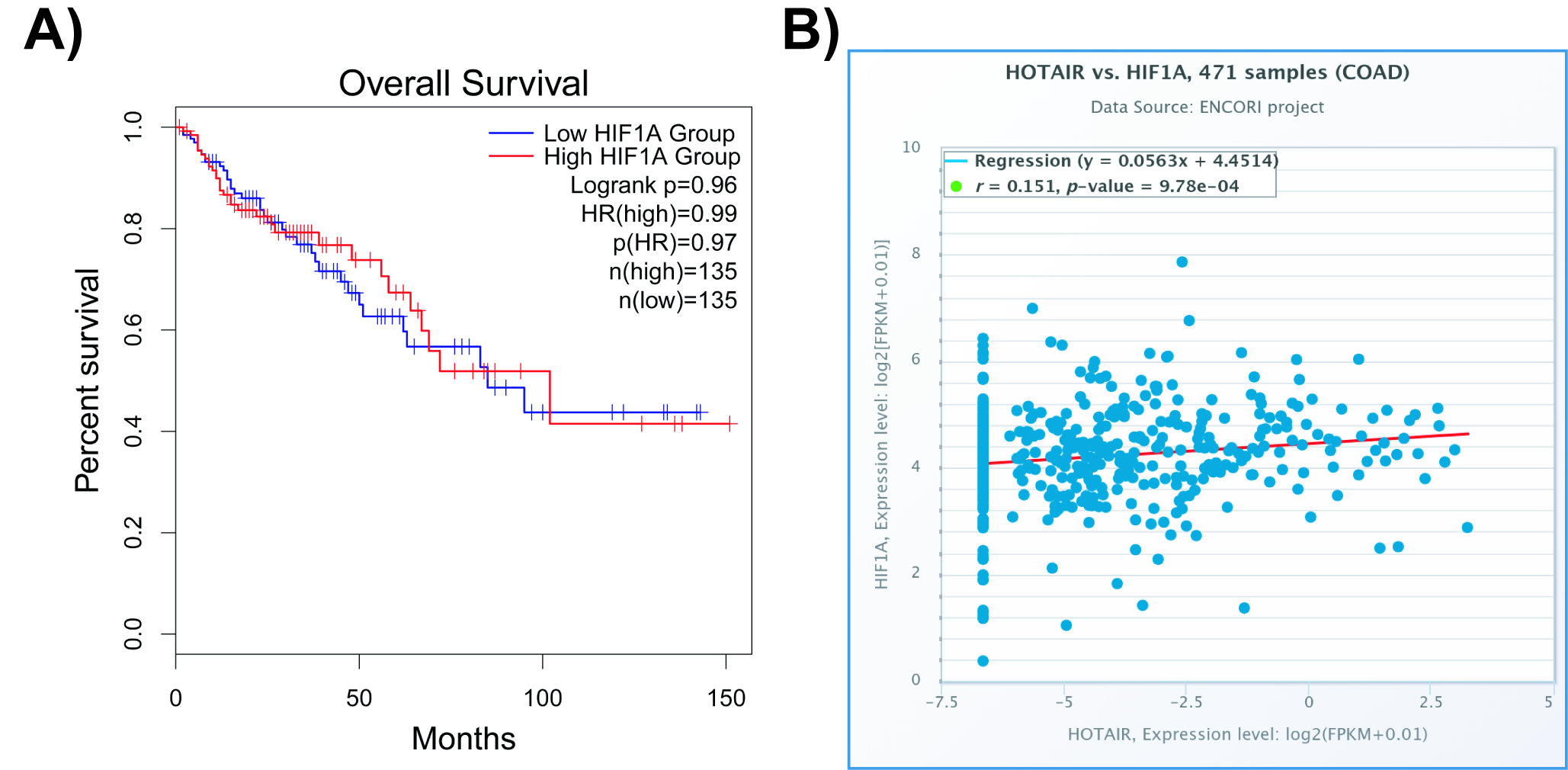

Supplement: Supplementary file 1 [file cells-14-00388-s001.zip › cells-3477114-supplementary/SUPPLEMENTARY FIGURE S1.tif]

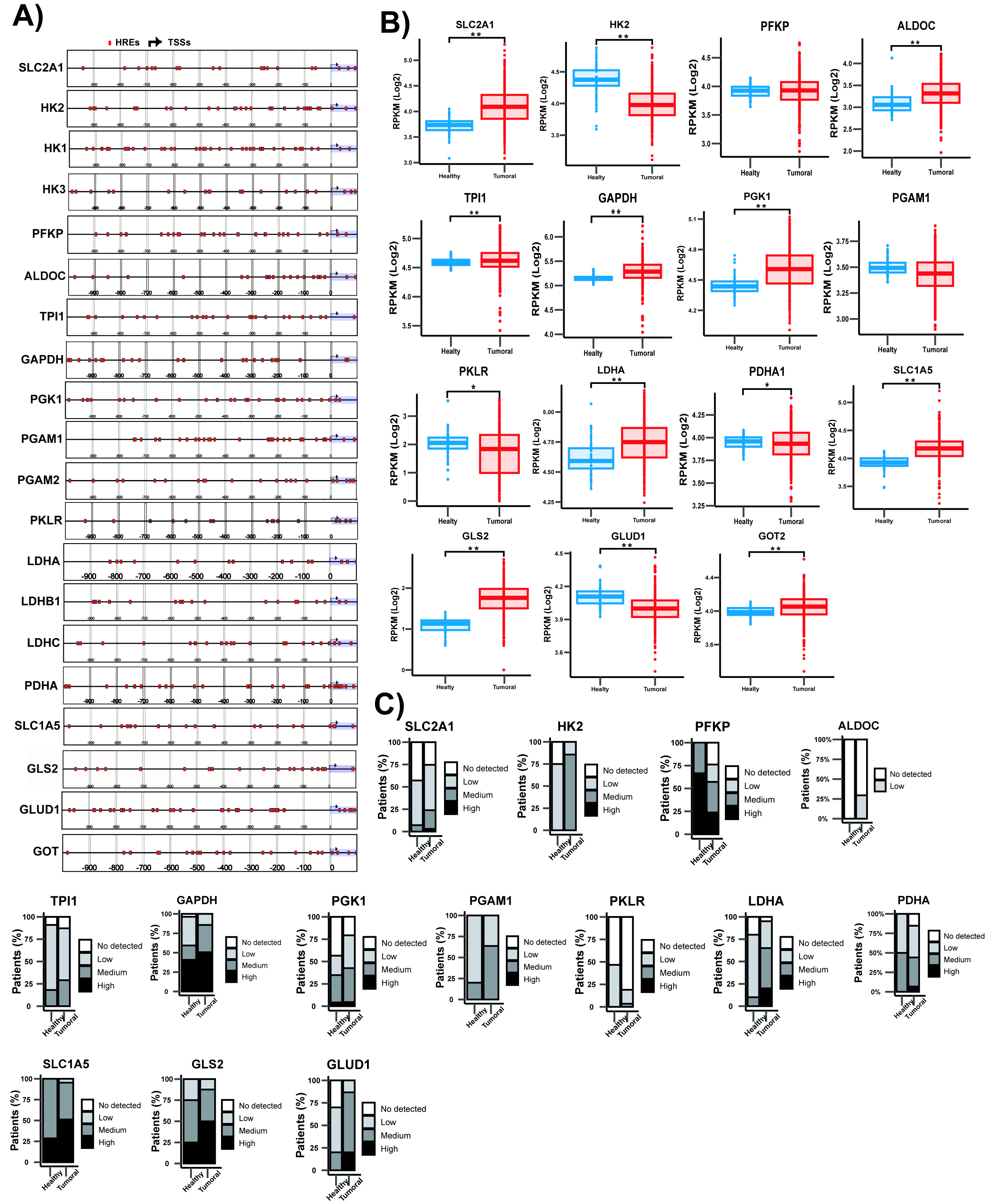

Supplement: Supplementary file 1 [file cells-14-00388-s001.zip › cells-3477114-supplementary/SUPPLEMENTARY FIGURE S2.tif]

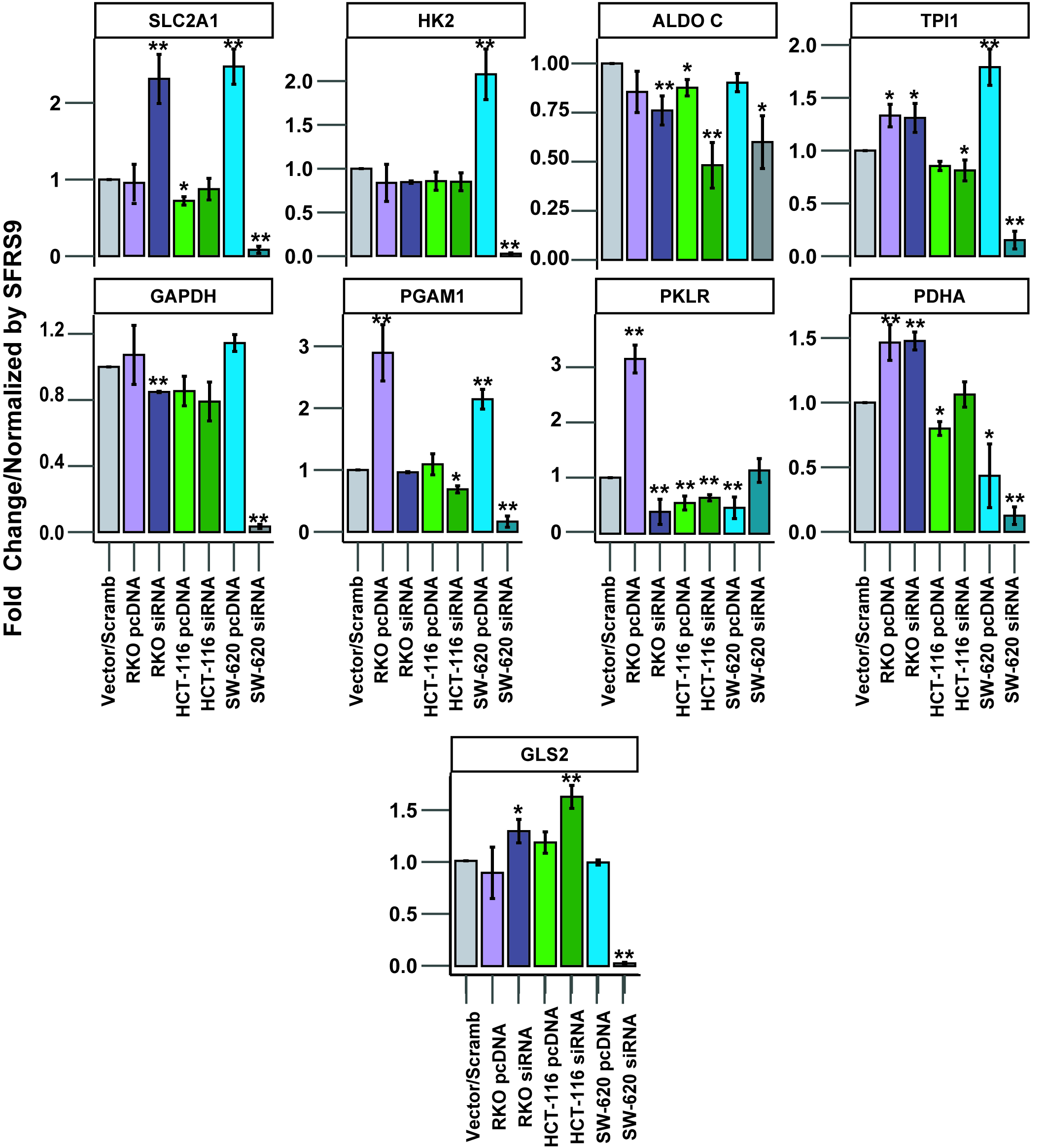

Supplement: Supplementary file 1 [file cells-14-00388-s001.zip › cells-3477114-supplementary/SUPPLEMENTARY FIGURE S3.tif]

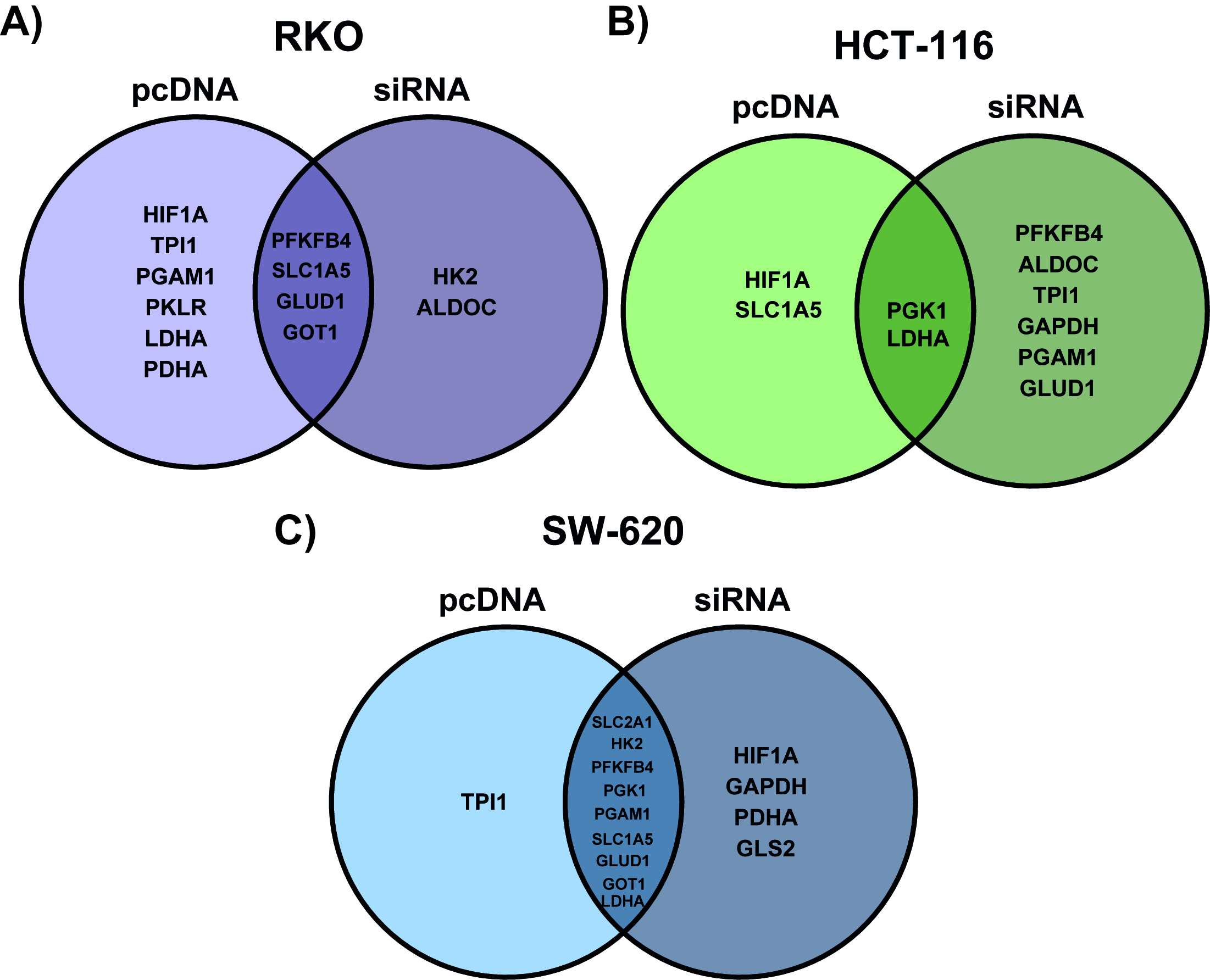

Supplement: Supplementary file 1 [file cells-14-00388-s001.zip › cells-3477114-supplementary/SUPPLEMENTARY FIGURE S4.tif]

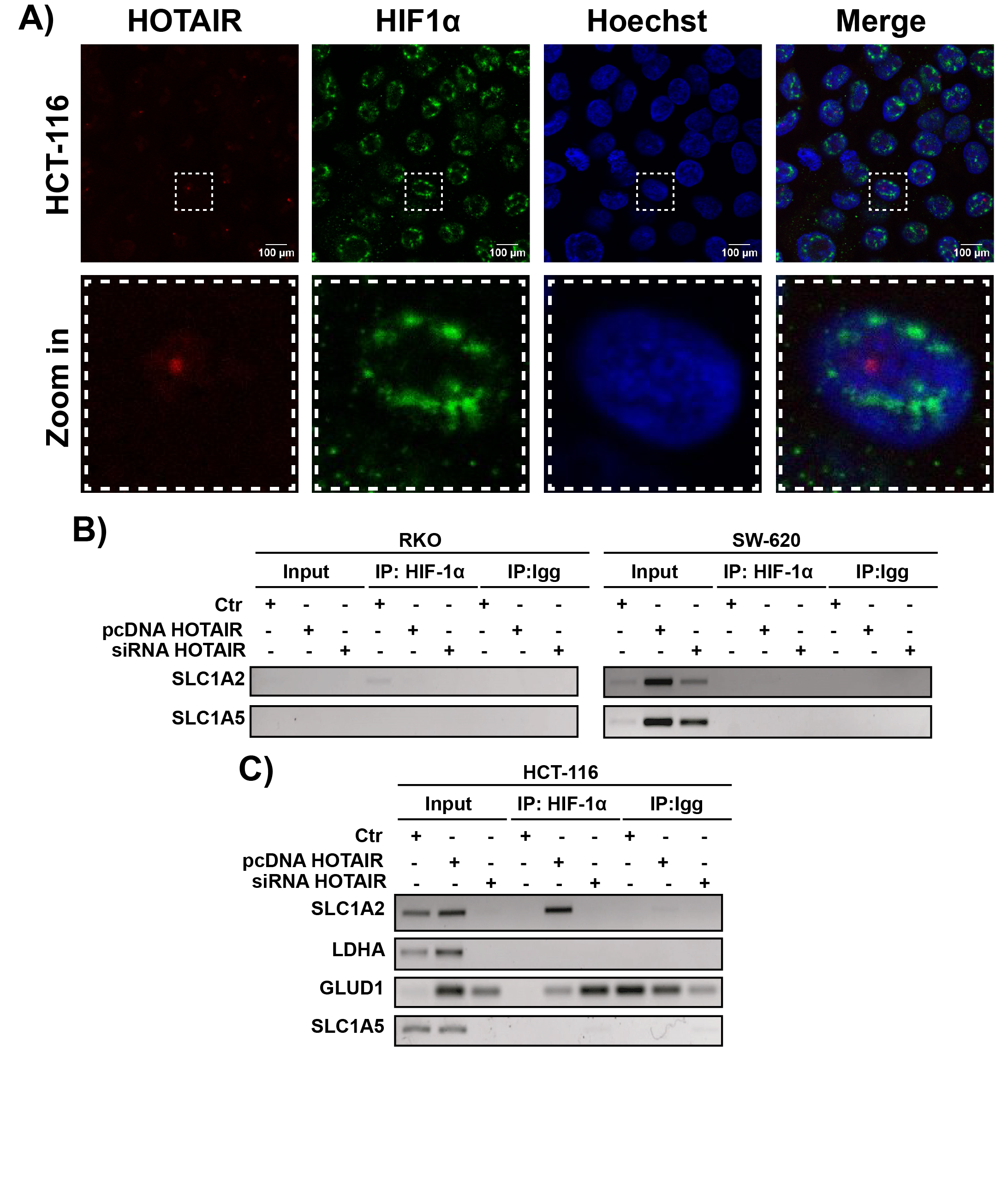

Supplement: Supplementary file 1 [file cells-14-00388-s001.zip › cells-3477114-supplementary/SUPPLEMENTARY FIGURE S5.tif]

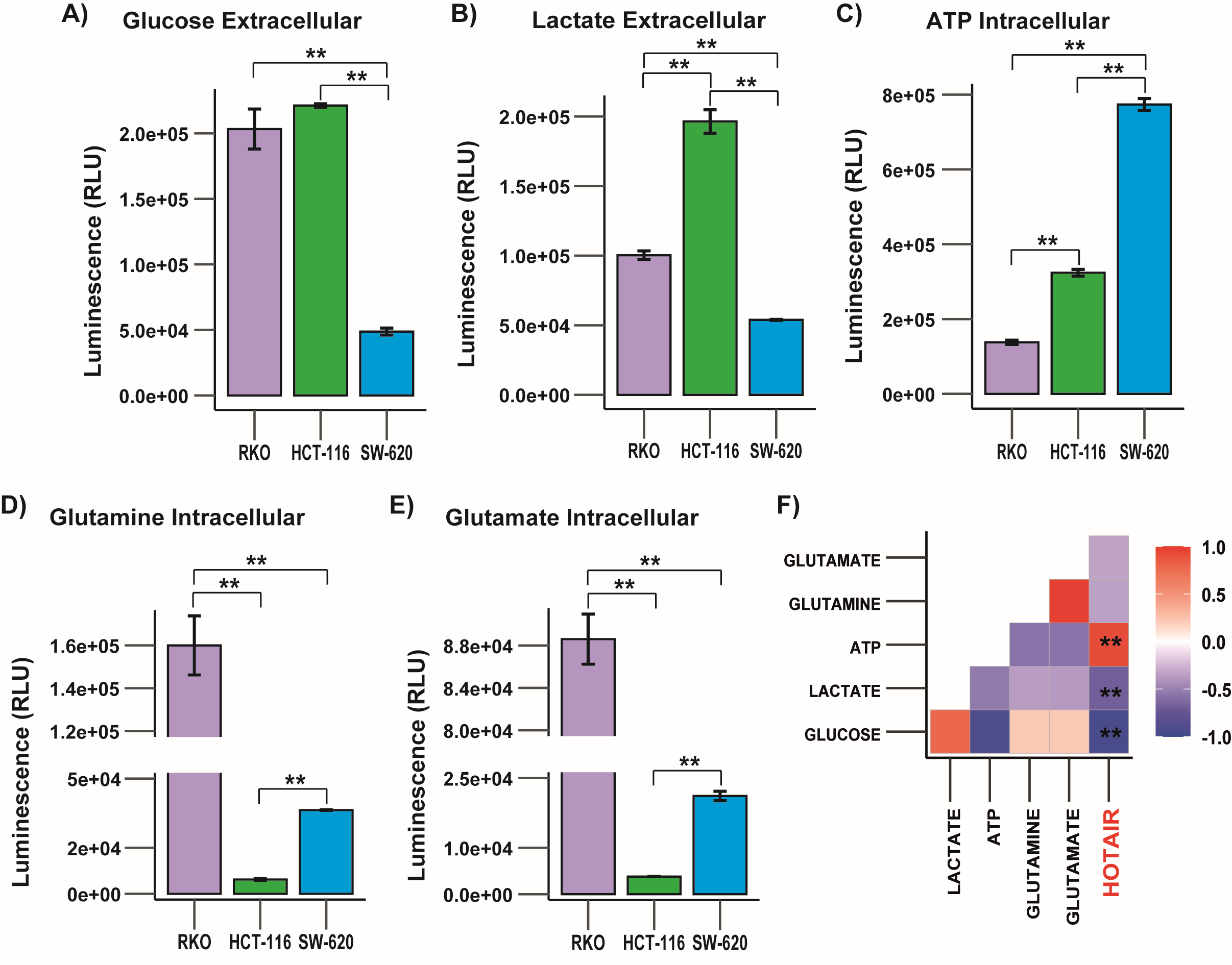

Supplement: Supplementary file 1 [file cells-14-00388-s001.zip › cells-3477114-supplementary/SUPPLEMENTARY FIGURE S6.tif]
